# Patients’ experiences of barriers and facilitators with continuous positive airway pressure therapy in obstructive sleep apnoea – a qualitative interview study

**DOI:** 10.1186/s13690-025-01645-w

**Published:** 2025-06-16

**Authors:** Karin Jeppesen, Anita Rabøl Good, Ilse Dall Dyrhaug, Marianne Bruun Johansen, Jette Primdahl

**Affiliations:** 1 Department of Otorhinolaryngology, Head and Neck Surgery, University Hospital of Southern Denmark, Sydvang 1, Sønderborg, 6400 Denmark; 2 Department of Regional Health Research, University of Southern Denmark, Odense, Denmark; 3 Hospital Sønderjylland, University Hospital of Southern Denmark, Aabenraa, Denmark; 4 Danish Hospital for Rheumatic Diseases, University Hospital of Southern Denmark, Sønderborg, Denmark

**Keywords:** CPAP, Apnoea-hypopnoea index, Semi-structured, Adherence, Sleep clinic, Qualitative content analysis

## Abstract

**Background:**

The prevalence of obstructive sleep apnoea ranges from 6 to 17% in the general adult population. Untreated obstructive sleep apnoea (OSA) is associated with cardiovascular disease, diabetes, traffic accidents, and decreased quality of life. Continuous positive airway pressure (CPAP) therapy is the gold standard for treating OSA. However, only approximately half of all treated patients succeed with this therapy. There is a lack of knowledge about the patient experience of CPAP treatment and the barriers and facilitators to successful CPAP treatment. The study aimed to explore how newly diagnosed patients with OSA experience receiving the diagnosis, their motivation for treatment, and essential factors for successful CPAP treatment.

**Methods:**

A qualitative design using individual semi-structured interviews was applied. Patients with successful (*n* = 10) and unsuccessful (*n* = 9) treatment were interviewed. Qualitative Content Analysis inspired by Graneheim and Lundman was used.

**Results:**

The analysis derived five themes: “Symptoms and thoughts about the diagnosis and CPAP treatment”, “Expectations and personal resources are important for success”, “The experience of problems and benefit from CPAP treatment”, “Social impact of CPAP treatment” and “Information and support from the sleep clinic.”

**Conclusions:**

Newly diagnosed patients with obstructive sleep apnoea describe barriers to successful CPAP treatment as lack of support from their partner and problems with the equipment. Important facilitators are positive expectations, partner support, and noticeable treatment effects. Early follow-up support, a biopsychosocial approach, and relatives’ involvement in the sleep clinic consultations are needed to support patients toward successful CPAP treatment.

**Table:** 

**Text box 1. Contributions to the literature**
• Studies have indicated that the experience of continuous positive airway pressure (CPAP) therapy poses a significant burden on individuals with obstructive sleep apnoea (OSA).
• Newly diagnosed patients with OSA describe barriers to successful CPAP treatment as lack of support from their partner and problems with the equipment, regardless of their adherence level.
• Important facilitators are an open attitude, positive expectations, partner support, and noticeable treatment effect.

## Background

Obstructive sleep apnoea (OSA) is an increasingly common condition, with repetitive pharyngeal collapse during sleep [1]. The prevalence ranges from 6 to 17% in the general adult population [2, 3], making OSA one of the most frequent diseases [4]. In Denmark, approximately 1,7% of the population is diagnosed with OSA [5], which indicates under diagnosis problems. Untreated OSA can lead to a significantly increased risk of morbidity and mortality [6, 7], and OSA is more likely to co-occur with obesity, structural abnormalities, diabetes, and cardiovascular diseases [8, 9]. The main symptoms of OSA are daytime sleepiness and fatigue, which can cause decreased quality of life, impaired cognitive function, increased risk of traffic accidents, and systemic hypertension [10, 11].

The diagnosis of OSA can be determined by a home sleep apnoea test, resulting in an apnoea-hypopnoea index (AHI). An AHI < 5 is considered normal. AHI of 5–15 is considered mild, AHI of 15–30 as moderate, and AHI > 30 as severe OSA [12]. Positive Airway Pressure (PAP or CPAP for Continuous) therapy is the primary treatment for patients with moderate to severe OSA [13]. If used consistently, CPAP is an effective treatment, but there is often limited and variable adherence among patients [14, 15]. The level of adherence is a measure of the patient’s consistent use of CPAP and is calculated based on how many hours the CPAP treatment is used in a given period. High adherence is defined as “use of CPAP treatment regularly for more than 4 hours/night for > 70% of the recorded period” [16]. Low adherence is defined as using CPAP for less than 4 hours/night for less than 70% of the nights in a given period. Non adherence refers to CPAP not being used in a given period. Studies report that 10–78% of patients achieve high adherence after initiating CPAP treatment. Thus, a large number of patients with OSA do not achieve successful CPAP treatment. However, the long-term adherence of CPAP is not well described [17–19].

Knowledge about the reasons behind variable short-term adherence and factors affecting long-term adherence from a user perspective is important for supporting patients in successful therapy.

Qualitative studies have found that CPAP treatment is experienced as a significant burden for patients with OSA [20–22]. In 2021, a systematic review and thematic synthesis was published on patient experiences of using PAP for OSA [23]. The analysis generated four themes: Journey to PAP, Discomfort from and around PAP, Adapting to and using PAP, and Benefits from PAP. The findings highlight the applicability of a biopsychosocial understanding of PAP use. As a limitation, they found that many of the included studies only recruited current PAP users and potentially reduced the inclusion of more difficult experiences of PAP treatment [23]. There is thus a need to gain insight into the patient’s experiences earlier in the process to improve support from healthcare professionals, increase the number of high-adherence patients, and achieve knowledge on patients’ experiences using PAP where PAP treatment is not yet established and successful. Cultural differences may affect clinical practice as well as the experience of PAP treatment, and only one of the studies was from Scandinavia [24]. Furthermore, only six of the included twenty-five studies were published during the past six years (2017–2019 [23]).

A scoping review investigating motivational interventions to improve CPAP adherence [25] found that motivational interventions were more effective compared to standard care despite the results not always being maintained over time. The authors recommend conducting qualitative studies investigating what type of information and strategies patients perceive as most meaningful. Such information could contribute to optimising motivational interventions for improved patient outcomes.

### Aim

This study aimed to explore newly diagnosed patients with OSA’s experience of (1) receiving an OSA diagnosis, (2) motivation for CPAP treatment, and (3) essential factors to succeed with CPAP treatment, including support from a sleep clinic.

## Methods

### Study design

We planned a qualitative study based on a hermeneutic approach using individual semi-structured interviews [26, 27].

### Setting and participants

The study was performed at the sleep clinic at Hospital Sønderjylland, University Hospital of Southern Denmark in Sønderborg, Denmark.

Patients diagnosed with sleep apnoea from a sleep registration (either in primary care or through the sleep clinic) evaluate their daytime sleepiness on the Epworth Sleepiness Scale (ESS). The ESS provides a sum score ranging from 0 to 24; the higher the score, the higher the person’s level of daytime sleepiness [28, 29]. The patients attend a medical examination at the sleep clinic to determine whether CPAP treatment should be initiated. If treatment is initiated, the patient receives CPAP equipment and guidance from an experienced sleep apnoea nurse. All patients were initially treated on an outpatient basis with auto CPAP, with a pressure range between 4 and 20 cm H_2_O. In Denmark, healthcare services are free and tax-financed regardless of the patients’ adherence to the treatment. After six to eight weeks, the patient meets for a follow-up consultation with an experienced nurse. All patients are offered follow-up appointments. Treatment is adjusted for patients with low or non adherence, and a shorter follow-up is scheduled.

Nurses in the sleep clinic acted as gatekeepers. The nurses informed eligible participants verbally about the study and offered written information. If, after time for consideration, the patients wished to participate, they provided written signed consent and arranged an appointment with the study nurse for an interview. The nurses invited ten patients with no or low adherence at the second consultation and ten patients with high adherence. To achieve varied information, we used stratified sampling to capture and provide information about experiences regarding CPAP treatment in the participants’ everyday lives. We aimed for maximum variation regarding age, sex, marital status (living alone or cohabitant), comorbidities, body mass index and level of adherence to ensure different perspectives [30]. Participants who did not speak or understand Danish were excluded. We used Malterud’s concept of information power to reflect on and guide the sample size [31].

### Data collection

An interview guide was developed based on the study aim, experiences of nurses and physicians at the sleep clinic and input from two patient research partners. Please see Table 1 for the overall themes in the interview guide.

**Table 1:** Interview guide used for semi-structured interviews on experiences with continuous positive airway pressure treatment for obstructive sleep apnoea

Themes	Follow-up questions
The diagnosis of sleep apnoea	What were you referred for? How did you experience receiving the diagnosis? What thoughts did you have about your future everyday life with sleep apnoea?
Handover of the CPAP machine	What did you already know about CPAP? What were your expectations for CPAP treatment? How did you experience the handover of the CPAP machine? What feelings did you have afterward?
Use of the CPAP machine	What challenges did you experience initially, and now after some time of use? What do you think is important for whether or not it is successful to use a CPAP machine?
Assistance from others for CPAP treatment	Do you have contact with others with sleep apnoea? Do you receive help/support for CPAP treatment from them? Has anyone reacted negatively to your CPAP treatment? Has anyone made a special difference for you in your sleep experience?
Quality of life - the experience of life before treatment and now	What has CPAP treatment meant for you? Do you experience its impact on your sexual/intimate life? How did you sleep before starting CPAP treatment? How do you sleep now?
Lifestyle changes, group dispensing, mentorship, and check-ups	Would you be interested in further assistance with lifestyle changes? Would you be interested in group dispensing of the CPAP machine and a mentorship program? What benefits do you see with both in-person and telemedicine check-ups?

Three sleep apnoea specialist nurses from the sleep clinic at Sønderborg Hospital performed semi-structured individual interviews with the participants. Each nurse did not interview participants with whom they had had a professional relationship from the clinic. Depending on the patient’s preferences, the interviews could be performed by telephone, online through Teams, or face-to-face in a quiet room at the sleep clinic.

All interviews were recorded. Secretaries from the Sleep clinic transcribed the interviews using a transcription guide to ensure consistency.

### Analysis

To describe the manifest and interpret the latent content of the transcribed empirical material, we used qualitative content analysis, as described by Graneheim and Lundman [26]. Table 2 describes the steps in the analysis. The transcribed interviews were assessed by at least two members of the research team (the first two steps in the analysis), and the entire author group assessed the condensing, coding, and development of themes. Table 3 provides examples of the analysis process.

**Table 2:** Steps in the qualitative content analysis of interviews on obstructive Sleeep Apnoea and continuous airway pressure treatment

Step	Description
1	Read all the transcribed interviews several times to capture a sense of the complete data material
2	Identify meaning units of importance to the aims of the study
3	Condense and code the identified meaning units on the manifest level on post-it notes including participant numbers. Adherent participants on a yellow post-it and non-adherent participants on red post-it notes.
4	Compare codes based on similarities and differences, and sort the codes into categories on wall posters describing the manifest content across all interviews
5	Based on the interpretation of codes and categories, overall themes are developed and described.

**Table 3:** Example of the analysis process from meaning units to themes in the content analysis of interviews on obstructive sleep Apnoea treatment experiences

Meaning unit	Condensed meaning unit	Code	Category	Theme
I am happy to speak about CPAP and there are more people than you realize that also have the device	Speaks openly about CPAP treatment	Openness to CPAP treatment	Openness and tabu	Symptoms and thoughts about the diagnosis and CPAP treatment
I had no idea what I was getting into, but it was enough to stop snoring as much and get a more peaceful sleep	No specific expectations, but expected less snoring and improved sleep	Expected less snoring and improved sleep	Expectations for CPAP treatment	Expectations and personal resources are important for success
My mask is simply so noisy. We’ve talked about sleeping separately, but we haven’t yet. We keep trying.	Mask-related problems can result in the need for a separate bedroom.	Noise from the mask leads to the need to sleep separately	The mask has an impact on the relationship.	Social impact of CPAP treatment

### Ethical considerations

Data collection and management was registered under the Danish Data Protection Agency at Hospital Sønderjylland, University Hospital of Southern Denmark (Journal nr.: 22/41839). We obtained written consent from the participants and data was stored at Hospital Sønderjyllands internal T-drive, which complies with the European General Data Protection Regulation and Danish law for data protection. The Regional Scientific Ethical Committees in The Region of Southern Denmark decided no formal approval was needed in accordance with Danish law as it was not a biomedical study (Journal nr.: S-2022000-104). No names, details, or sensitive information are reported to ensure confidentiality and conceal the participants’ identities.

## Results

We included ten adherent and nine non-adherent participants. Table 4 reports socio-economic and disease-specific information for the participants. The participants in the adherent group had BMI of median 25 (range 23–47), AHI median 29 (range 20–35) and ESS median 7 (range 3–12) while participants in the non- or low adherent group had BMI median 27 (range 25–38), AHI median 34 (range 14–60) and ESS median 11 (range 2–17). The interviews were conducted by telephone from September 2022 to November 2022. None of the interviews were conducted online or face-to-face.

**Table 4:** Demographic and clinical characteristics of patients with successful or unsuccessful continuous positive airway pressure treatment for obstructive sleep Apnoea

Adherent participants							
Age* (years)	**Sex**	Adherence to CPAP*	AHI before start of CPAP*	**ESS**	BMI*	**Civil status**	**Work situation**
70–74	F	80–89%	Severe	8	18,5–24,9	Co-habitant	Retired
45–49	M	80–89%	Moderate	6	> 30	Co-habitant	Full-time
70–74	M	80–89%	Severe	12	25–29,9	Co-habitant	Retired
50–54	F	90–100%	Severe	4	-	Single	Full-time
45–49	F	80–89%	Severe	10	> 30	Single	Part-time
65–69	M	90–100%	Moderate	3	25–29,9	Co-habitant	Retired
60–64	F	90–100%	Moderate	4	25–29,9	Co-habitant	Full-time
35–39	M	70–79%	Moderate	8	25–29,9	Co-habitant	Full-time
35–39	F	80–89%	Severe	11	> 30	Co-habitant	Full-time
65–69	M	80–89%	Moderate	5	18,5–24,9	Co-habitant	Retired
**Non- or low-adherent participants**							
30–34	M	0–9%	Severe	17	> 30	Co-habitant	Full-time
70–74	M	0–9%	Moderate	7	25–29,9	Co-habitant	Retired
60–64	M	30–39%	Severe	12	25–29,9	Co-habitant	Full-time
45–49	F	30–39%	Mild	4	25–29,9	Co-habitant	Retired
45–49	M	30–39%	Severe	NA	25–29,9	Co-habitant	Full-time
60–64	M	10–19%	Mild	17	25–29,9	Co-habitant	Full-time
60–64	M	50–59%	Severe	9	25–29,9	Co-habitant	Full-time
45–49	M	50–59%	Severe	2	25–29,9	Co-habitant	Full-time
60–65	M	30–39%	Severe	13	> 30	Co-habitant	Retired

* To ensure participant pseudonymity, selected variables that could be particularly identifying have been grouped

*F*: Female; *M*: Male; *CPAP*: Continuous Positive Airway Pressure; *AHI*: Apnoea-hypopnoea index < 5 normal, 5–15 mild, 15–30 moderate and > 30 severe; *BMI*: Body Mass Index < 18,5 underweight, 18,5–24,9 normal, 25–29,9 overweight and > 30 severe overweight; *ESS*: Epworth Sleepiness Scale ranging from 0 to 24, the higher the score, the higher the person’s level of daytime sleepiness; *NA*: Not Available

The analysis derived five themes: “Symptoms and thoughts about the diagnosis and CPAP treatment, “Expectations and personal resources are important for success”, “The experience of problems and benefit from CPAP treatment”, “Social impact of CPAP treatment” and “Information and support from the sleep clinic”. In the following, each theme is described in more detail and illustrated by selected quotes.

### Symptoms and thoughts about the diagnosis and CPAP treatment

Participants described frustrations and worries after being diagnosed with OSA and before starting CPAP treatment.

*“I associated sleep apnoea with elderly overweight men*,* so it was tough mentally” (male 30–34 years – adherent)*.

However, many participants were happy to receive a diagnosis, as they learned about the reason for their current symptoms and the treatment options. It was also important for them to reduce the risks of untreated sleep apnoea.

*“I was disappointed about the disease or diagnosis*,* but something could be done - I could come home with a solution under my arm” (male 45–47 years – adherent)*.

Some participants who had prior knowledge about OSA from family, friends, colleagues, and the media reported fewer frustrations/worries before treatment.

*“Not really worried because I already knew a little about it from my former boss and my brother” (male 60–64 years – low/non-adherent)*.

The most frequent symptoms the participants described that led to diagnosis were fatigue, snoring, and breathing problems. In addition, participants had been diagnosed with OSA due to lack of energy and decreased memory - often women. Participants referred for evaluation of sleep apnoea due to snoring or breathing problems were almost all men. The participants described how a partner or others had noticed snoring and/or breathing problems. However, some participants had experienced waking up with feelings of not being able to breathe. For others, partners were alerted and nervous about the lack of breathing and were disturbed by the loud snoring.

*“I snore*,* and my wife was nervous about my lack of breathing at night” (male 70–74 years – low/non-adherent)*.

In addition to fatigue, snoring, and breathing pauses, several participants also described other symptoms that led to evaluation for OSA, such as headache, heart disease, and the feeling of “heavy eyelids”.

### Expectations and personal resources are important for success

The participants described how their expectations and personal resources affected their motivation for treatment. The patients had positive but varying expectations for the treatment and its effect. However, a general expectation was that their fatigue and snoring would be treated/reduced. This expectation was a significant motivation for starting with CPAP.

Several participants felt that the prospect of their symptoms and comorbidities either improving or ceasing was an important motivating factor.

*“If sleep apnoea has an impact on my blood pressure and CPAP can make me reduce my medication*,* that would be a good thing” (male 60–64 years - low/non-adherent)*.

Some male participants described the importance of attitude and openness to ensure CPAP treatment success. Others described that the greatest motivation was feeling the effect of the CPAP treatment and experiencing a positive change in their daily lives.

Many of the participants from both groups reported that support and conversations about CPAP treatment with their relatives, employers, and social circle positively affected their motivation for the treatment. In addition, some were motivated as CPAP treatment changed the symptoms and thus their concerns about the partner/family.

*“I think it’s (swear word) more or less done for my wife’s sake*,* so she could get some proper sleep” (male 45–49 years - low/non-adherent)*.

Some participants described how starting CPAP treatment was a big adjustment since they could not sleep well with the equipment every night, and some nights not at all. In addition, they felt it was demotivating to continue the treatment.

*“I expected to feel more rested and fresh during the day. The first night was really good*,* and I was so refreshed during the day. The second night was bad; I was frustrated and upset*,* it went up and down” (female 35–39 years - adherent)*.

Only one participant expressed no knowledge about CPAP treatment or support from a social network. However, several participants also described feelings of insecurity. They found it challenging with many new practical issues and worries.

### The experience of problems and benefits from CPAP treatment

It was difficult for the participants to get used to the CPAP treatment. Some needed several months of adjustment, partly due to problems with falling asleep, interrupted sleep, discomfort from mask/tube, gas in the stomach, dryness in the nose/mouth/throat, heat, and feelings of suffocation.

*“For the first few months*,* I would wake up and press the on/off button [to reset the regulation of pressure]*,* and it took a while before I fell asleep again. Now I give it [the button] a tap*,* and then I fall asleep again” (female 45–49 years - low/non-adherent)*.

Some described quickly experiencing better sleep, feeling more rested, and noticing a difference after the first night of treatment. These participants reported improved quality of life after initiating CPAP treatment.

Mask problems were initially a major issue for most participants, resulting in mask changes. Many found it challenging to choose the most suitable mask because they were unfamiliar with the range available and whether they needed a nasal or full-face mask. Likewise, they lacked knowledge about what it was like to sleep with a mask on at night. They doubted whether a mask was appropriately fitted, as they had had nothing with which to compare. Mask leakage was one of the predominant complaints. The causes were, among other things, facial hair and lack of experience in adjusting the mask.

*“It “farts” when you sweat and blow up your eyes” (female 45–49 years – adherent)*.

This resulted in sores on the bridge of the nose, noise and leakage, limited sleeping positions, and restless and interrupted sleep. Several male participants, but none of the female participants, described their partners being bothered by the sound and air from the mask.

Many of the participants experienced positive effects of CPAP treatment, such as more restful sleep, fewer nocturnal toilet visits, increased energy in daily life, increased desire for physical activity, better memory, and a sense of a better quality of life. Some also reported that CPAP treatment had reduced several of their other symptoms, such as headaches/migraines, restless legs, snoring, and fatigue.

*“I feel like the light bulb has been fully plugged in now (female 45–49 years - adherent)*.

A group of participants also felt that CPAP treatment had little or no effect. They described unchanged or increased fatigue. Despite this, none expressed a desire to discontinue CPAP treatment. Several attributed their motivation to continue due to their knowledge about the consequences of untreated OSA.

*“The motivation is the risks of untreated sleep apnoea because I‘m a little disappointed with the effect on my fatigue” (male 65–69 years - adherent)*.

Despite describing the minimal treatment effect, some reported that their partners experienced the opposite: more energy in the participant.

### Social impact of CPAP treatment

Many participants reported that CPAP treatment positively impacted their relationship with their partner. For example, they could now sleep together again as snoring was no longer a disturbance, and their evening energy levels were higher.

*“Now I can sleep with my wife again*,* something I haven’t been able to do for almost 1.5 years due to snoring” (male 60–64 years - low/non-adherent)*.

Other participants described the treatment as mentally challenging, affecting intimacy. They felt restricted in physical contact and closeness.

*“It’s tough. I don’t put on the mask until the lights are out. I don’t want to lie close to my husband with the mask on*,* I don’t want him to come near me when I have it on” (female 35–39 years – adherent)*.

Other participants described limitations with other relatives to CPAP treatment, as the mask was perceived as scary for children or grandchildren.

Some participants with no partner described having doubts about introducing the CPAP machine to a future partner.

*“New partner - I don’t think it makes the chances better*,* but I also think that I used to snore a lot*,* which wasn’t nice. This is a better alternative” (male 30–34 years – low/non-adherent)*.

Some participants expressed feeling the diagnosis of OSA and CPAP treatment was taboo.

*“It’s nice that there were others who knew about it*,* but I would also like to keep it to myself - I don’t think it’s anyone else’s business what diseases I have or don’t have” (male 30–34 years – low/non-adherent)*.

Almost all participants with good adherence described being open about their CPAP treatment with their family and friends. In the participants with low/non-adherence, openness about CPAP treatment was more varied. About half did not want or did not feel the need to talk to their family/friends about CPAP treatment. Some saw challenges in staying overnight outside and/or with others.

### Information and support from the sleep clinic

The participants shared their experiences from the first visits to the sleep clinic regarding the professional support and information they had received and future ways of using support to succeed with the CPAP treatment. Generally, the participants assessed that the information they received at the clinic was sufficient and informative during medical and nursing consultations. Some described that it provided reassurance, while others emphasised the nurse’s and doctor’s ability to be positive, answer relevant questions, make practical suggestions, and take time for the individual, such as demonstrating sleep data on a PC screen and providing visual demonstrations by the doctor of the anatomy of the throat. All of the above factors impacted the initiation of treatment.

Topics not addressed during the initial conversation but important to participants included the long-term aim for CPAP treatment and the consequences of untreated sleep apnoea. Some participants wished they had received more information about how difficult it could be to adjust to CPAP treatment. Others were unsure if they had received the desired information or had forgotten it, such as about comorbidities, driver’s license regulations, cleaning, mask selection, adherence requirements, and tele-monitoring.

Despite the valuable information, many found the situation overwhelming, and some described that they could not process more information. Other critical comments included feeling that the consultation with the doctor was too fast and that they were just a number in a queue.

Many of the participants were positive about the possibility of group consultations but not necessarily keen to participate themselves. Likewise, the participants had widely different desires for the composition of such a group. Some wanted to meet others of the same age and gender, so they were with like-minded people. Others thought the challenge would be the same whether they were 20 or 60 years old, in the workforce or not. However, everyone agreed that the group size should not exceed six people.

*“Groups would not make a difference to me*,* of course*,* there may be some questions from other patients that you haven’t thought of yourself” (male 65–69 years - adherent)*.

Participants with good adherence said they did not want or feel the need to participate in groups. Reasons included that it would be crossing a boundary, fear of not being heard or appearing dumb, keeping intimate questions to themselves, and exposing themselves.

*“No one will ask dumb questions*,* no one will attract attention*,* no one will appear dumb*,* no one will ask embarrassing questions*,* no one will say what do I do when I want to have sex” (female 50–54 years - adherent)*.

In the first period after initiation of CPAP treatment, the participants were positive about telephone and email contact with the sleep clinic, where it was easy for them to get in touch and receive quick responses. Many participants described these communication channels as a “lifeline” combined with the information from the distributed brochures.

At the follow-up check after 6–8 weeks, participants were satisfied with the opportunity to refresh healthcare information, ask additional questions after treatment initiation, and have the machine and mask reviewed again. Participants described these as factors that impacted whether treatment succeeded.

In general, most participants preferred physical consultations compared to virtual/phone contacts as they felt they would get more out of seeing and discussing their development in treatment sitting across from a healthcare professional rather than on a screen. Participants preferring virtual check-ins highlighted the benefits of avoiding the drive and taking time off work. However, virtual contact was only considered relevant when the treatment worked and there were no problems to discuss and only if the patients still had an opportunity to come into the sleep clinic in case of problems.

*“Attendance is definitely an advantage*,* I had some things that needed adjusting*,* I want to see and hear the people I need to explain it to” (male 45–49 years - adherent)*.

Despite no specific questions during the interview, several participants focused on the need for other treatment options to CPAP. In particular, they inquired about treatment with dental appliances, weight loss, and possible surgery.

*“Of course*,* I had hoped that I could just have surgery*,* but that was not the case” (male 30–34 years*,* low/non-adherent)*.

## Discussion

This study aimed to explore newly diagnosed patients with OSA’s experience of (1) receiving an OSA diagnosis, (2) motivation for CPAP treatment, and (3) essential factors to succeed with CPAP treatment, including support from a sleep clinic. The patients experienced frustration, concern, hope, and relief when receiving the OSA diagnosis. Initially, motivation for treatment was good for most, as they hoped their OSA symptoms and the risk of comorbidities would be reduced. Both the adherent and low/non-adherent groups of participants agreed that the most important facilitators for successful CPAP treatment were an open attitude, positive expectations, noticeable treatment effects, and support from family and the sleep clinic.

A common issue across the derived themes was motivation. Participants described knowledge of the OSA disease, expectations of treatment, and risks of comorbidities with untreated OSA as motivating factors for ensuring treatment worked. Therefore, it is important to address these issues early in the treatment process to optimise the motivational interventions for improved patient outcomes [25]. Participants who experienced an effect of CPAP treatment, such as less fatigue, became even more motivated to ensure CPAP treatment was successful. Conversely, participants who did not immediately experience the effect of treatment had a more challenging time finding the motivation to become “friends” with the CPAP device. Practical challenges such as problems with the mask, hose, and machine could also contribute to lower motivation, as seen in a previous qualitative study [32]. To obtain support in case of practical challenges, patients need easy access to help from a sleep clinic, such as through phone or online contact.

For participants, it was imperative to have support from their partner, and many described how the CPAP device affected their relationship, and for some, it had some negative consequences. The role of spouses/partners in initiating patients’ CPAP treatment has been described in previous studies [33] and investigated in a scoping review from 2022 [34], with 21 studies, of which five were qualitative [20, 23, 35–38]. The review concluded that the support of the relatives, especially at the beginning of the treatment, is essential for the patient’s use and motivation for CPAP treatment. The involvement of relatives will provide the relatives with a better foundation to support and motivate the patient and may help reduce possible concerns and resistance to the CPAP treatment. Furthermore, treatment of OSA with CPAP can have health benefits for spouses/partners too [39]. Collaborative partner involvement may be a useful strategy for increasing CPAP adherence. Therefore, healthcare professionals could consider involving relatives at the start of treatment. For example, encouraging patients to include a relative at the first consultation in the sleep clinic and allowing the relatives’ concerns and issues to be addressed in the consultation. If they cannot attend physically, it may be possible to give them online access if the patient agrees. Patients without relatives may need additional support from the sleep clinic staff to ensure adherence.

Despite participants reporting that they feel well-informed about the treatment, several indicated that they would have liked more information about the length of the adaptation period. Others suggested that an extensive amount of information should not be given simultaneously. Health literacy is the ability to read, understand, critically appraise, and use health-related information [40]. A Danish study found that between 14.5% and 18.3% of adults find it challenging to follow health instructions, ensure that healthcare providers understand their problems correctly, and feel unable to discuss their health concerns with healthcare providers [41]. A patient’s health literacy level is associated with self-management ability in chronic diseases [42]. Thus, we expect that low health literacy will also affect the ability to succeed with CPAP treatment among patients with OSA.

Sleep clinics could consider splitting the information session in half and providing video podcasts and written information as supplements. In addition, it may be relevant to plan earlier follow-up support after initiation of treatment, i.e. after two weeks as suggested in recent literature [43] so that the patient can report start-up problems and treatment can be adjusted before the problems grow too big. This follow-up could be virtual [44] unless patients are experiencing problems with the mask and need another type. Follow-up support should target the patient’s health literacy level to reduce the number of patients with low or no adherence. The difference in participants’ attitudes towards attendance or virtual check-ups reflects the need for more individually tailored follow-up, too.

Our expectation for the study was that the group’s experiences with good adherence would differ from those with poor adherence. However, there were surprisingly many similarities in their experiences and thoughts about the OSA diagnosis and starting CPAP treatment. There was a clear tendency for participants with good adherence to be open about their illness, whereas participants in the non-adherence group were often more closed and/or did not think others should be involved. This observation also expresses the enormous taboo that still exists in society about OSA, which some participants described as a social burden.

The reason for poor adherence to CPAP in patients with OSA is multifactorial [45] and it is well established that both very young adults and patient over the age of 80 are less likely to achieve good adherence to CPAP treatment compared to middle-aged patients [46, 47]. Thus, it is important that we, as healthcare professionals, address the whole patient, not just the practical management of the CPAP device and the biomedical aspects. As described in a previous study, a biopsychosocial understanding is needed. There should be opportunities to discuss the psychosocial factors that can constitute barriers to treatment success with the health professionals [23]. This includes the patient’s feelings about diagnosis, partner’s attitude and reactions, openness about the treatment, concerns related to CPAP treatment, and feelings if treatment fails. It should also involve information about where and how the patient can receive support when facing problems.

### Strengths and limitations

One of the strengths of this study is that the participants include patients who have successfully started CPAP treatment and participants who found it problematic and did not achieve high adherence. In addition, we included participants of different ages, relationship statuses, comorbidities, and body mass index, so the interviewed group reflects the population we see daily in the sleep clinic. Furthermore, it is a strength that the author group consists of doctors and nurses with extensive knowledge in the field and nurses and researchers from other specialties, so data was not only interpreted from a discipline-specific perspective. Finally, we assessed that we had achieved sufficient “information power” with 19 participants. There was not much new information in the last 2–3 interviews, but it may be a limitation that there was only one woman in the low adherence group despite striving for similar variety in sex between groups.

### Perspectives

This study has helped us gain knowledge about the barriers that patients struggle with in their daily lives to make the treatment successful, and the clinical opportunities to include psychosocial areas in the dialogue, tailor the information to the individual participant’s needs and health literacy level, and develop follow-up support options for patients with low or non-adherence. Future studies could explore whether implementing a biopsychosocial approach, involvement of relatives and different approaches to information and follow-up can contribute to successful CPAP treatment for more patients.

Our study was not designed or powered to detect statistical differences between patients with good adherence and those with no or low adherence. However, descriptive data on BMI, AHI, and ESS scores indicate that median values are higher in the group with no or low adherence. Future research should investigate whether this association is significant.

We found that family members significantly impacted participants’ motivation, and future studies could examine whether non-adherent patients’ motivation is influenced by having a supportive partner, a partner who dislikes the CPAP device, or perhaps no family member who can support their treatment. In these cases, healthcare providers may offer special follow-up care to this specific patient group.

## Conclusions

When receiving an OSA diagnosis, patients experience frustration, concern, hope, and relief. Initially, their motivation for treatment is good, as they hope that their OSA symptoms and the risk of comorbidities will be reduced. Regardless of adherence level, patients with OSA describe barriers to successful CPAP treatment as lack of support from their partner and problems with the mask, hose, or machine. The most important facilitators for successful CPAP treatment are positive expectations, personal resources, an open attitude to tell others about the treatment, noticeable treatment effects, and support from family, the broader social network, and the sleep clinic. Early follow-up support, a biopsychosocial approach, and relatives’ involvement in the sleep clinic consultations are needed to support more patients toward successful CPAP treatment.

## Data Availability

The datasets generated and analysed during the current study are not publicly available in accordance with Danish law and due to personal information in the interviews. Data are available from the corresponding author on reasonable request.

## Abbreviations

BMI: Body mass index
CPAP: Continuous positive airway pressure
ESS: Epworth sleepiness scale
OSA: Obstructive sleep apnoea
PAP: Positive airway pressure
AHI: Apnoea-hypopnoea index
